# Responding to the public health consequences of the Ukraine crisis: an opportunity for global health diplomacy

**DOI:** 10.7448/IAS.18.1.19410

**Published:** 2015-03-17

**Authors:** Tim K Mackey, Steffanie A Strathdee

**Affiliations:** 1 Department of Anesthesiology, University of California, San Diego School of Medicine, San Diego, CA, USA; 2 Division of Global Public Health, University of California, San Diego School of Medicine, San Diego, CA, USA; 3 Global Health Policy Institute, San Diego, CA, USA

**Keywords:** health diplomacy, syndemic, health policy, international relations, foreign affairs, opioid substitution therapy, Ukrainian conflict, harm reduction

## Abstract

**Introduction:**

Peace and stability in Eastern Europe is now at a crossroads with the rapidly deteriorating foreign policy crisis continuing to unfold in the Ukraine. However, largely overlooked in the context of other foreign policy and diplomatic priorities are the serious public health consequences for the region following the annexation of Crimea and the subsequent decision to ban opioid substitution therapy in the disputed territory.

**Discussion:**

On 1 May 2014, the Republic of Crimea officially announced it would end access to opioid substitution therapy, an essential harm reduction tool recognized by international organizations and virtually all other European countries. The policy development marks a critical reversal in the region’s fight against its growing HIV epidemic and also threatens years of public health gains aimed at providing evidence-based and integrated treatment approaches to combat drug dependence and HIV. Beyond these risks, the Ukrainian conflict could also negatively impact control of other infectious diseases that are converging with HIV and injection drug use, such as multidrug-resistant tuberculosis and hepatitis C virus. The continuing conflict is also likely to have a significant negative impact on Ukraine’s fragile public health system leading to even worse population health outcomes than currently experienced by the country.

**Conclusions:**

In response to this crisis, the application of global health diplomacy principles represents a possible route of advocacy to ensure that HIV prevention, humane treatment of substance using populations, and improving public health outcomes in the region are pursued among concerned international stakeholders. In order to be effective, global health diplomacy efforts must be coordinated and advocated in all forms of diplomatic engagement, including at the core, multistakeholder and informal levels and through existing channels such as the different human rights bodies of the United Nations as well as amongst other actors. Hence, the Ukraine crisis represents a critical moment for the practice and advancement of global health diplomacy in order to ensure global public health priorities are given their rightful place in foreign policy making to hopefully help in bringing resolution to the current conflict.

## Introduction

In a recent piece in the *BMJ*, Michel Kazatchkine, the UN Secretary General’s Special Envoy for HIV in Eastern Europe and Central Asia, brings needed attention to the negative public health consequences arising from the current regional conflict in the Ukraine [1]. Specifically, Kazatchkine highlights how the rapidly deteriorating political and foreign policy crisis in the Ukraine, Russia and disputed annexed territory of Crimea could have long-term consequences for scientifically based public health interventions aimed at addressing the epidemic of injection drug use and HIV in Eastern Europe [1]. However, beyond these concerns raised by Kazatchkine’s article, ongoing destabilization also has the potential for broader regional public health implications. This includes the risk of greater HIV transmission over porous borders, convergence of HIV with synergistic diseases of tuberculosis (TB) and hepatitis C virus (HCV) among people who inject drugs (PWID), and continuing weakening of Ukraine’s public health infrastructure [2–4] .

The challenge posed by this ongoing regional conflict also marks a crucial opportunity to prioritize public health concerns in ongoing foreign policy and diplomatic efforts by concerned nation states. This includes the potential application of an emerging form of diplomatic statecraft known as “global health diplomacy” (GHD), generally defined as diplomatic activities focused on issues of global health importance and prioritization of global health issues in the foreign policy context [5,6]. In this commentary, we expand on the Kazatchkine’s discussion by exploring the unique public health threats associated with the current Ukraine–Russia conflict and how the practice and application of GHD could lead to benefits for public health, regional political stability and shared global health security.

## Discussion

### Public health impact of conflict

On 1 May 2014, the Republic of Crimea officially announced it would end access to opioid substitution therapy (OST) following its controversial declaration of independence and subsequent annexation by Russia [1]. Though a public referendum in the territory to join Russia received an affirmative vote, it has largely been criticized as violating Ukraine’s constitution and international law [7]. The annexation has also not been recognized by the larger international community, with the UN General Assembly (UNGA) adopting a non-binding resolution declaring the referendum as invalid and annexation by Russia illegal [7].

Crimea’s politically based decision to end OST post-annexation resulted from the Ministry of Health of the Russian Federation and drug control authorities specifically directing the Crimean government to adopt this policy. It also represents policy spill-over from the Russia ban of OST supplies to the region and Russia’s own draconian domestic policy that prevents medical use of OST (methadone and buprenorphine maintenance) for treatment of opioid dependence [1]. Despite HIV/AIDS representing a continuing and escalating public health concern domestically, attempts to change Russia’s national policy on harm reduction practices and specifically OST have not been effectuated and may be influenced by powerful and entrenched stakeholders that directly oppose OST for non-scientific reasons [8].

Russia’s annexation of Crimea has resulted in OST now being deemed illegal in the territory, potentially undermining several years of Ukrainian public health gains aimed at reversing its rapidly escalating HIV epidemic [1]. The immediate effect of this pronouncement means that needed drug dependence treatment for an estimated 800 Crimean clients is now inaccessible, some of whom now look to leave the region to find treatment elsewhere or suffer forced withdrawal symptoms [1]. Additionally, reports of Crimean clients being imprisoned and/or returning to drug use, with some suffering death from suicide or complications related to drug overdose and chronic illness, highlight the immediate human toll of this politicized issue [9]. The significant challenges that would be faced by reinstituting OST in post-conflict areas like Crimea are also ongoing concerns, as well as the potential that the ban could spread to other territories in dispute between the Ukraine and Russia.

OST is endorsed by the World Health Organization (WHO), Joint United Nations Programme on HIV/AIDS (UNAIDS), and UN Office of Drugs and Crime (UNODC) and also recognized as an essential medicine and intervention (both methadone and buprenorphine are WHO “essential medicines”) [10]. Global support of OST is steadily rising, with OST available in over 60 countries worldwide, including all the members of the European Union [11]. Indeed, Russia’s policy ban means it is the only Council of Europe member state where OST treatment is unavailable, representing a significant departure from national policies aimed at ensuring quality drug treatment access in the rest of the region [11]. This is despite the fact that the effectiveness of OST in reducing risk factors associated with injection drug use and HIV transmission are well established, viewed as cost-effective, and have been largely adopted by the broader international public health community [10].

## Syndemic potential and other public health concerns in the region

In addition to the loss of OST access for an estimated 14,000 people covered by Crimea’s HIV prevention programmes, the control of other infectious diseases converging with HIV and injection drug use in the region could also be impacted [1]. Particularly, the potential threat of an infectious disease “syndemic” (defined as: “the convergence of two or more diseases that act synergistically to magnify the burden of disease”) requires heightened concern [4]. The current conflict could also worsen Ukrainian population health outcomes that have been adversely impacted by past geopolitics, including a historically low life expectancy and high morbidity/mortality rates [12].

The potential for the region to experience a syndemic of converging infectious diseases of HIV, TB and HCV has not been fully studied. However, outbreaks of multidrug-resistant tuberculosis (MDR-TB) have ravaged Russia’s prison systems and increased to record levels in other regions of the country [13,14]. Given that PWIDs comprise the most important group at risk for both HIV and TB in Russia, a MDR-TB and HIV syndemic due to Russia’s growing political isolation from the international community and disruption of public health technical assistance is a concerning possibility [15,16]. Add to this convergence a “hidden” epidemic of HCV among PWID and the viruses’ association with strict drug policies such as Russia’s OST ban, elevates the potential of a possible HIV-TB-HCV syndemic if not properly monitored [17]. Hence, the possibility of convergence of HIV, MDR-TB and HCV among PWIDs, in an environment with the highest number of new HIV infections in Eastern Europe, could be exacerbated by the end of OST in the now Russian controlled Crimean peninsula [1].

Additionally, more fundamental concerns regarding the stability of Ukraine’s public health and healthcare systems are being worsened by a conflict that has wreaked havoc on the country’s already fragile economy [12,18]. With one of the lowest life-expectancies (68.8) in Europe, a decreasing Gross National Income (22% decrease from 1990 to 2012), and a below average Human Development Index (0.740) compared to other countries in Europe and Central Asia, Ukraine was already facing significant economic and health challenges prior to the current conflict [19]. These problems also manifest in an “east–west gradient” where populations in the western region (mostly Ukrainian-speakers) experience lower mortality rates than their eastern counterparts (mostly Russian-speaking) [12]. Collectively, these challenges exacerbated by past and current political destabilization/transition, point to the need for immediate multilateral and diplomatic support for healthcare policy reform and international development aid to prevent a collapse of Ukraine’s health systems [12].

## Crimean OST ban: defying international consensus

Despite limited priority in foreign policymaking, advocacy attempting to address the Ukraine crisis’ impact on public health has commenced. This includes a written appeal from a broad coalition of various NGOs and academics led by The International Network of People who Use Drugs (INPUD), addressed to representatives of the United Nations and its specialized agencies (including The UN Special Rapporeur on right to health and Special Rapporteur on torture and other cruel, inhuman or degrading treatment or punishment, UNAIDS, UNODC, WHO, UN Development Programme, and UN High Commissioner for Human Rights (OHCHR)). The urgent appeal calls for international pressure against Russia’s unilateral actions and multisectoral efforts to ensure ongoing OST access for affected patients [20]. It also argues that Russia’s OST ban in Crimea constitutes a violation of international human rights law and the right to access essential medicines (including violating Article 12 of the International Covenant on Economic, Social and Cultural Rights (ICESCR)) [20].

Additionally, the OHCRC, in investigating potential human rights abuses in the Ukraine, has also reported that some OST provisioning healthcare facilities were being controlled by armed groups significantly limiting treatment access [21]. It also found that availability of drugs and treatment was negatively impacted by security issues and disruption in public transportation and that clients were returning to drug use with the accompanying risks of increased cases of HIV and HCV infections, all representing possible violations of the human right to health [21]. In response, NGOs such as the International HIV/AIDS Alliance in the Ukraine, supported by funding from the International Renaissance Foundation and Elton John Foundation, have initiated special humanitarian projects in an attempt to ensure uninterrupted supply of OST for Crimean clients [22].

These developments contrast starkly with Ukraine’s own departure from Russia’s OST ban beginning in 2004, when the country embarked on the design, piloting, implementation and scale up of OST, syringe exchange programs, and other evidence-based approaches for dealing with drug dependence and HIV [23]. These efforts also coincided with Ukrainian development of integrated care services for HIV, TB and drug dependency positively highlighted by a 2010 WHO report, though these advances may be at risk if escalation of the conflict cannot be averted [24].

At the same time, as the situation continues to escalate, Russia is increasingly becoming isolated from the international community politically and economically. This includes the ousting of Russia from diplomatic venues such as the G8 group of industrialized countries, cancellation of the G8 Summit planned in the Russian city of Sochi, withdraw of its voting rights by the Council of Europe, and suspension of negotiations over Russia joining the Organisation for Economic Co-operation and Development and the International Energy Agency, all in direct response to its annexation of Crimea [25]. These exclusions brings with them, along with harsh sanctions and a projected Russian economic recession, the possible end of decades-long cooperation in science and public health, including joint efforts by USA and Russia that led to the development and production of polio vaccine, eradication of smallpox and bilateral aid including US supply of vaccines and pharmaceuticals in assisting with infectious disease outbreaks in Russia [26,27]. The Crimean annexation has also led to a halt in “science diplomacy” initiatives (generally defined as cooperation in science to improve international relations/foreign policy) between the two countries, including a threat to end cooperation on the International Space Station and a US Department of Energy ban on research exchange between US and Russian scientists [16].

Russia’s continuing policy entrenchment in banning OST is also seemingly contradictory in the larger context of a growing international movement supporting harm reduction practices [28]. This includes the UNODC, which recently announced groundbreaking recommendations through a key working group explicitly stating that criminal sanctions are not beneficial for addressing drug dependence [29]. This reversal in a long-standing policy of supporting enforcement-based approaches by the international drug control regime represents a strong move in support of harm reduction approaches and further demonstrates international consensus in direct opposition of Crimea/Russia’s OST prohibition.

## Science, harm reduction, and HIV diplomacy: a call for GHD action

Despite the high stakes for public health, substance abuse, HIV and other infectious disease control in the current Ukrainian conflict, health policy concerns have been largely overlooked in the current diplomatic process when compared to other traditional foreign policy priorities such as trade, economics, energy, politics, sanctions and defense [1]. Hence, though GHD represents recognition in foreign policy circles (including among diplomats, policymakers, public officials, and other stakeholders) that global health issues (such as globalization of infectious diseases) are growing in importance and influence in diplomatic negotiations and outcomes, its application to the Ukraine crisis has yet to adequately materialize [30]. Though some may debate whether traditional foreign policy domains continue to drive diplomatic decisions that impact public health decisions/policy, or conversely if foreign policy decisions are now being substantially influenced by global health priorities, this difference seems trivial given the need for urgent diplomatic action to protect population health in the entire region [2,31,32].

**Figure 1:**
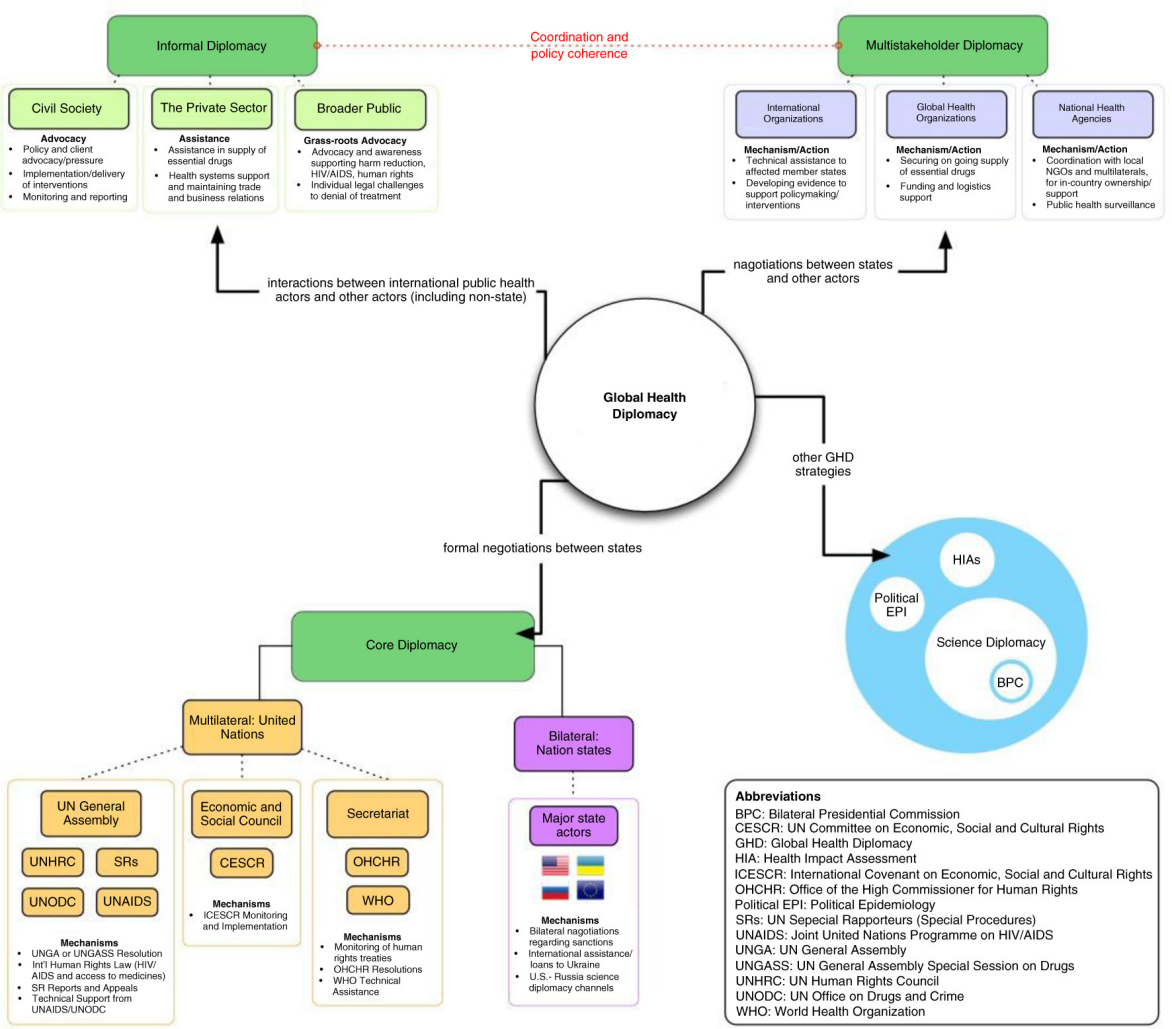
GHD strategies for the Ukraine crisis. Visual summary of stakeholders, channels and mechanisms.

Instead, the ongoing regional conflict and OST ban in Crimea are clear examples of where the principles of GHD should be utilized to achieve shared goals of global health security, improve the national economy, promote global infectious disease control and ensure the fundamental right to health. However, in order for GHD to be effective, health priorities arising from the conflict need to be advocated across a broad array of stakeholders and the different categories of diplomatic interactions between them (including through formal bilateral and multilateral channels, trade and economic sanction negotiations, alliance building, conflict resolution/peace-building discussions and civil society driven advocacy) [2]. This includes emphasizing the severe public health repercussions of the OST ban, the potential for syndemic outbreak in the region, and the need to support Ukraine’s flailing public health system through different channels of GHD. Diplomatic channels that need to be engaged include “core diplomacy” (formal bilateral and multilateral negotiations and agreements between and among nation states), “multistakeholder diplomacy” (negotiations between nations and other actors including partnerships among government agencies with multilateral institutions) and “informal diplomacy” (interactions between international public health actors and a host of other actors including non-state actors including agreements with donor, academic and humanitarian agencies) [2,5,33]. See Figure 1 for a summary of GHD proposed stakeholders, channels and mechanisms.

Specifically, this GHD strategy can be pursued at the “core” level through robust multilateral negotiations amongst nation states in international fora that are already engaged on the issue and/or those who generally advocate for OST. This specifically includes human rights bodies of the UN, such as the Human Rights Council (UNHRC) and UN Special Rapporteurs (both under the UNGA), the Committee on Economic, Social and Cultural Rights (CESCR) (under the Economic and Social Council) and the OHCHR (under the Secretariat and the International Covenant on Economic, Social and Cultural Rights). As argued by the INPUD coalition, UN member states have well-established international legal obligations not to directly contravene the fundamental human right to health, a principle that is clearly in jeopardy for thousands of HIV infected and substance using individuals. Additionally, numerous UN resolutions issued by the UNGA, UNHRC and OHCHR (including the Political Declaration on HIV and AIDS) have already reaffirmed international commitment in reducing vulnerability to HIV/AIDS, preventing discrimination for HIV patients, and ensuring access to essential medicines and HIV prevention programs. The Crimean OST ban represents an assault on all of these internationally agreed upon principles, as it violates the health rights of Crimean clients and likely endangers the health of others in the region, and also directly impedes global efforts to ensure HIV prevention, access to treatment, and non-discrimination.

On this basis, concerned UN member states should work through the existing UN human rights apparatus with the goal of securing a UNGA resolution reaffirming existing human rights obligations in the context of HIV prevention and treatment that are directly negatively impacted by the current conflict and calling for all parties (including Russia and Ukraine) to ensure that their actions and policies do not contravene population health outcomes and the fundamental human right to health. A good starting point for diplomacy would be CESCR and the Special Rapporteur on torture and other cruel, inhuman or degrading treatment or punishment (SRT). Specifically, CESCR has already expressed concerns regarding Russia’s OST ban within the context of ICESCR implementation and has called on the country to apply a human rights-based approach to drug use (including supporting access to OST, needle/syringe exchange programs and overdose prevention programs) [34]. Similarly, the SRT has raised concerns that a state-based OST ban could constitute a form of ill-treatment or torture of drug users given its physical and psychological toll, as well as it constituting abusive treatment and unjustified discrimination based solely on health status [35]. Broader UN support could also form the basis for a future UNGA or UN General Assembly Special Session (UNGASS) on Drugs resolution recognizing the broader importance of declaring harm reduction strategies and supporting OST access as a fundamental component of preventing infectious disease transmission to ensure collective global health security, especially in the context of political, economic and social disruptions such as the conflict in Ukraine [28].

Additionally, bilateral negotiations at the core level amongst key state actors are also a critical component of the GHD strategy outside of multilateral settings. This includes insertion of public health issues and priorities into any current or future negotiations between the US, the European Union, Ukraine and Russia on facilitating the peace process through easing of crippling trade and economic sanctions that have been worsened by the failing price of oil and the depreciation of the rouble [36]. Particularly, the lifting of the OST ban in Crimea or adoption of a position of non-enforcement by Russia of its ban in the territory could be made a condition of any sanction-related negotiations. In tandem, the bilateral foreign policy agenda and delivery of aid by the US and European Union (including sensitivity to protecting public health resources in any required structural reform from bilateral loans and aid) should not neglect the need to support progressive and sustainable reforms for Ukraine’s public health system with the overall goal of improving current deficiencies in population health outcomes [12]. This investment in public health could lead to improved security and economic development in the long-term [12].

At the multistakeholder-level, steps can be taken by international organizations (WHO, UNAIDS, UNODC), global financing and procurement institutions (e.g. the Global Fund) and national health agencies that have endorsed OST, to enter into formal and non-formal relationships with affected state and local governments and health ministries to strengthen public health capacity and ensure continuation of OST access for affected populations. This could include efforts to raise funds for OST and HIV prevention services, encourage needed technical assistance for affected areas/treatment sites, provide support for client relocation, and help secure ongoing supply of essential OST drugs. Strategies designed to encourage broader multistakeholder GHD and international cooperation should also be combined with bilateral support from country health agencies and other civil society actors.

Multistakeholder channels should also coordinate with informal channels of GHD aimed at direct advocacy and raising awareness regarding the benefits of OST among all concerned stakeholders including country officials, civil society, NGOs, the private sector and the broader public [2]. This should include targeted policy advocacy to promote acceptance of harm reduction practices in Russia and Crimea among policymakers and constituents, emphasizing its cost-effectiveness, the evidence-base supporting its use and combating existing social stigmatization of drug users and HIV/AIDS populations, all factors that have been identified as barriers to OST acceptability/implementation [8]. This can specifically include targeted support for localized NGOs already active in the region, including the INPUD, Eurasian Network of People who Use Drugs, HIV/AIDS Alliance of Ukraine and the International HIV/AIDS Alliance, to name a few.

GHD strategies should also include generating policy priority around the clear risks of HIV and infectious disease spread if evidence-based tools are suspended due to *political* instead of public health reasons [1]. Emerging concepts such as “political epidemiology,” which seeks to understand political determinants of health, can and have been used to explore how government policies preclude populations from evidence-based HIV prevention services and could be pursued in conjunction with a formal independent Health Impact Assessment (HIA) examining the negative health consequences of the OST ban in order to produce evidence-based policy recommendations in support of diplomatic negotiations and advocacy [37]. The evidence generated from such activities could also form the basis for additional evidence and support for legal complaints filed by Russian heroin addicts suffering from HIV and HCV that have challenged their denial of OST therapy in different venues (including Russian courts and the European Court of Human Rights) [38,39].

Finally, bilateral mechanisms that have facilitated decades-long cooperation between the US and Russia in the form of science diplomacy should also be leveraged. This specifically includes shared opportunities for public health advances and diplomacy, especially in the area of new therapeutic innovations for control of infectious diseases. Possibilities include US–Russian health assistance on technology transfer for accessing new rapid TB screening and diagnostic tools critical for case management and treatment and new direct-acting antivirals for the treatment of chronic HCV infection, both of which could help Russia in addressing its growing TB/MDR-TB and HCV problem [40,41]. To effectuate cooperation, existing bilateral channels, such as the Bilateral Presidential Commission established by President Barack Obama and then Russian President Dmitry Medvedev in 2009, need to be reinvigorated and used as a shared avenue for policy and diplomatic synergy between concepts of GHD and science diplomacy, with an emphasis on promoting the benefits of harm reduction practices as a critical component of controlling infectious diseases [27].

## Conclusions

The real threat of losing the fight against HIV in Eastern Europe, the possible growth and spread of MDR-TB and HCV converging with HIV among PWID, and the need for the international community to coalesce around supporting evidence-based harm reduction practices and Ukraine’s struggling public health system provide a critical moment for GHD to come to action. This requires putting public health concerns at the forefront of the current Ukraine/Russia crisis, and ensuring that foreign policy actors recognize the importance of global health issues on the broader international economy, global health security, and regional political stability.

Though health diplomacy is growing in theory and practice among academics and a small cadre of diplomatic health attachés, the current circumstances in the Ukraine require tangible action to rightfully advance this form of “smart” diplomacy to ensure the safety and health of global populations [33]. Coincidentally, a 2013 Carnegie Endowment report on Health Cooperation in US–Russian Relations summed up the unique opportunities of health diplomacy best by stating: “Health diplomacy is the great leveler which brings countries together in common cause, fighting one of humanity’s most ancient and powerful foes: disease” [26]. It also serves as an important reminder of why GHD principles need to be promoted and exercised across multiple stakeholders to advance the urgent priorities of population health in areas of 21st century conflict such as the Ukraine and Crimea.

As the conflict escalates and other regions of the Ukraine, such as Donetsk, are influenced by pro-Russian separatist elements and move closer to secession, the stakes for public health only increase. Hence, the crisis unfolding in the Ukraine represents an opportunity and indeed requires the active practice of GHD across the broad spectrum of the international community to ensure that the prevention of HIV and protection of the right to health takes its rightful place at the centre of foreign policy decisions.

## Author information

Tim K. Mackey is the Director of the Global Health Policy Institute, the Associate Director of the Joint Masters Program in Health Policy and Law at UC San Diego, and an Assistant Professor at UC San Diego School of Medicine in the Department of Anesthesiology and Division of Global Public Health. Steffanie A. Strathdee is the Associate Dean of Global Health Sciences, Harold Simon Professor and Chief of the Division of Global Public Health in the Department of Medicine at the UC San Diego School of Medicine.
